# Advanced optical measurement techniques for simultaneous fibre orientation and flow analysis complemented by machine learning

**DOI:** 10.1038/s41598-025-25656-3

**Published:** 2025-11-07

**Authors:** Tim Vaupel, Florian Gerland, Thomas Schomberg, Olaf Wünsch

**Affiliations:** Department of Fluid Dynamics, University of Kassel, Kassel, 34125 Germany

**Keywords:** Engineering, Materials science, Optics and photonics, Physics

## Abstract

A new measuring method is presented that allows time-resolved quasi-simultaneous measurement of fibre orientation and flow velocity of a transparent fluid such as a substitute fluid for fresh concrete or polymer melt, thus enabling the flow-related fibre orientation process to be visualised and thus better understood. In order to study individual fibres and their orientation in detail a PIV-based measurement stand was built, which is capable of analysing the flow and the fibre orientation in the suspension quasi-simultaneously. To measure the fibre orientation, black light can be switched on so that the fibres are stimulated by phosphorescence and become visible in the fluid. A random forest algorithm is used to detect the fibres in the images. This machine learning method allows the fibres to be accurately detected with little training data and a short training and processing time. The results show that there is a strong orientation effect on the fibres the closer they are to the orbit in the vicinity of the interfering body. In addition, a rapidly occurring orientation can be determined in detail. This allows the velocity vector fields and the actual fibre motion to be combined and analysed in this work.

## Introduction

Understanding and modeling of fibre-flow and fibre-fibre interactions is still a field of research that is complex to model due to the limited availability of experimental data especially on the micro scale, which makes it challenging to validate and refine existing models.

The approach presented here shows the measurement of a transparent substitute fluid in which the movement of tracer particles and fibres is measured simultaneously. This ultimately allows the flow and fibre movement to be determined to better understand their interaction. Fibre orientation, especially with fibre-fibre, fibre-particle and fibre-wall interaction, is difficult to model and therefore difficult to analyse numerically. Further difficulties are that most fluids such as fresh concrete or polymer melts are non-transparent and therefore optical measurements can only be carried out on the surface.

The objective of this measurement method is to be able to precisely measure the flow behaviour and the above-mentioned interactions. This should improve our understanding of the flow behaviour and orientation of fibres in complex suspensions. The new measurement method is applied to the experimental investigation of a flow around a cylindrical interfering body in a rotating container. This use case meets a very widespread application in that a variety of fresh concrete rheometers use similar geometries.

**Fig. 1:**
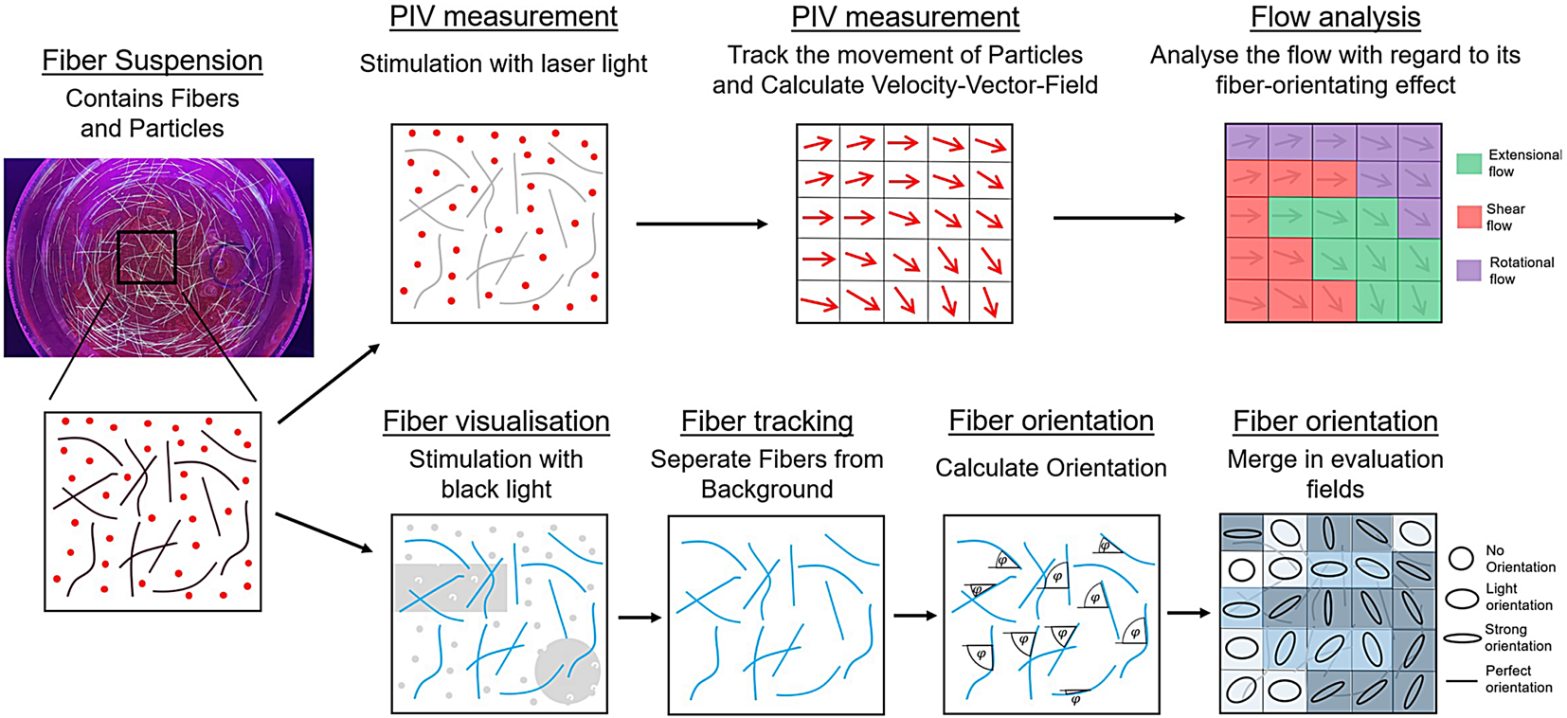
Graphical abstract of the presented method. The methodical path from the experiment to the evaluation of the flow conditions and the fibre orientation is shown.

The first part of the paper deals with the construction of the test rig. Subsequently, the flow is investigated with the PIV system and analysed with suitable methods with regard to the fibre-orientating effect of the flow. Finally, fibre tracking is carried out using a random forest classifier and the orientation is examined in detail using orientation tensor analysis as shown in the graphical abstract in Fig. 1.

### Preliminary work

One of the most widely used measuring devices for suspensions such as fresh concrete, mortar or other particle suspensions are the ball probe rheometer and the cylinder probe rheometer. These measuring devices make it possible to measure the flow properties of suspensions with particles and fibres. The design of the experiment is based on these devices. (See Fig. 3)

The flow and the resulting fibre orientations in a ball probe rheometer were numerically investigated by our research group in earlier work^15–17^. Flow curves for the Bingham-Plastic model and the fibre orientation for low fibre volume fractions, for fibre geometries and concentrations that satisfy the Folgar-Tucker model and exhibit negligible fibre-wall and fibre-fibre interactions were obtained. However, sufficient experimental validation, analysis of fibre-fibre and fibre-wall interactions and general model validation are lacking. This work can add scientific value in terms of flow analysis, fibre visualisation and orientation analysis, and fibre tracking using AI methods.

### Scientific fundamentals

This work combines topics from the fields of materials science, rheology and computer vision using artificial intelligence (AI). For this reason, the respective fundamentals are addressed in detail in this chapter.

#### Measurement systems

In the construction sector, the most widely used material class is cement or concrete, which consists of a wide variety of particles with different particle sizes and shapes, additives and, for fibre-reinforced materials, also different fibres (shape, length, diameter, flexibility). A common method of measuring flowability in the construction industry is the funnel flow time (according to DIN EN 12350-8, DIN EN 1015-3, DIN EN 12350-12), which is used to categorise the suspension into flow classes without using objective material parameters (F1-F6 according to DIN EN 206-1/DIN 1045-2 C0-C3)^40^.

To measure the flowability scientifically, relative measurement systems are used such as the falling ball viscometer^41^, the Vane rheometer, the Tattersall rheometer and the ball probe rheometer (Fig. 3). They have been developed to analyse the flow properties of suspensions with fibres^9,19,24,28,42^.. In the work of Bibbo^5^ and in the work of Marin^27^, Martinie^26^ and Férec^12^, a change in the measured viscosity can be determined as a function of the fibre volume fraction. The fibre orientation is measured implicitly in all these measuring systems. At the beginning of the measurement, an increased viscosity is usually measured (if the fibre volume fraction is sufficient). This decreases as the measurement progresses until it remains in a quasi-stationary state. The fibre orientation process is measured here. Unoriented fibres in the liquid generate higher shear stresses, which decrease as the orientation of the fibres progresses and ultimately remain in an oriented state^26^.

Determining the fibre orientation in detail the micrograph method is used. This examines the hardened component, which is cut open in several planes. The cut surfaces can then be analysed with regard to the fibre orientation^4,32^. One non-destructive method is to locate the fibres using computer tomography (CT). The fibres must consist of a material that is visible on the CT images. The fibre orientation can be determined three-dimensionally using a large number of images from different directions, whereby this method is mainly used in solidified materials^23,29,36^.

The determination of fibre orientation in the flow is very challenging and has only been investigated or successfully performed in a few studies to date^11^. It is especially difficult to measure the fibre orientation while the process is going on, which however promises insightful information for the modeling itself.

#### Flow visualisation Methods

The PIV method (Particle Image Velocimetry) is used to analyse flows by adding tiny particles to the fluid. These are illuminated with a laser (in a laser cut) and recorded using camera(s). Using the cross-correlation method (or similar), the particle movement is processed into a vector field using image pairs. The following points must be taken into account:

- Good following behaviour of the particles to the fluid
- Transparency of the liquid (air, water, silicone oil,...)
- Transparency (refractive index matching) of fibres/other particles in the liquid
- Avoid/calculate laser reflection and refraction
- Avoid sedimentation/lift of the particles
- Suitable particle size
- Suitable volume fraction of the particles

Tracking at high flow velocities is possible with a high clocking of the laser and the camera^1^ at various flows^7,18,20,37^. The movement of larger particles and fibres can also be used to calculate flow velocities from image recordings. The rotational motion of the non-spherical particles can also be detected^37^.

#### Fibretracking with random forest

**Fig. 2:**
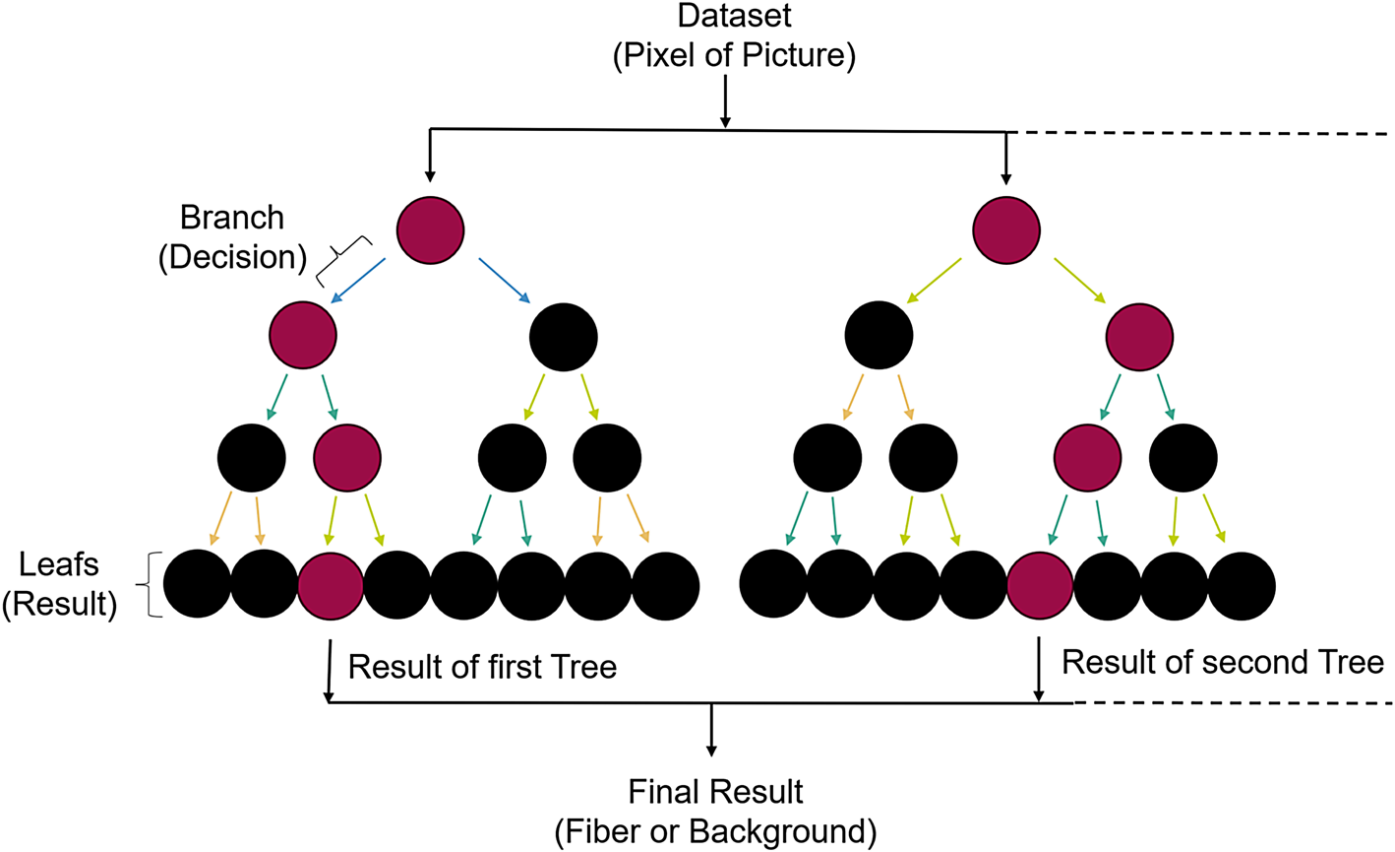
The basic structure of a random forest algorithm. Decisions are made with the features of the respective trees, which are ultimately summarised in an final result.

Random forest is a common machine learning method that is used in object recognition. It is used for classification and regression tasks. It is an ensemble method, which means that a large number of individual results are summarised to produce a final result. It is a supervised learning method, which means that input data and output data are linked together during the learning process. A training data set must be provided to the algorithm for learning. This consists of images with the information about where the fibres are located in the training image^21,22^. The basic idea of the algorithm is to divide the logic into the ‘forest’, the ‘tree’, the ‘branches’ and the ‘leaves’. The ‘forest’ consists of individual trees, which are referred to as decision trees. A ‘tree’ consists of ’decision branches’ that lead to a result (end of the tree/‘leaf’). See Fig. 2.

Questions must be created as a basis for the decisions of the branches. The questions are not asked in a fixed order and are not necessarily based on all previously defined features. Based on the training data set, the algorithm learns which questions (‘branches’) must be asked in which order that the result can be predicted with the highest possible probability. The features that are required to create the questions must be defined in advance of the training. These features can be properties such as colour, brightness, gradient to neighbouring pixels, a local binary pattern or other properties. A large selection of features usually leads to better results. However, it must be taken into account that the computational effort can increase considerably when using a large number of features and/or features that are complex to calculate. The challenge here is to select the most important features in order to combine a short computing time with a good result.

Each ‘tree’ is trained on a random subset of the data set, which is also known as the bagging method. This means that there are as many sub-models (trees) as there are subsets of the data set. Each sub-model does not necessarily have to deliver trustworthy results, but the number of ‘trees’ produces a stable result from the sub-models. In order to prevent correlation of the decision trees, different splits of the training data set are usually used (cross validation).

Our team is not aware of any explicit application of a random forest algorithm in fibre tracking. However, there are many areas of application in which it has been successfully established. An example that used this algorithm successfully is from Smith^33^, who applied it in the determination of bacteria. A Random Forest Algorithm was tested in comparison to applied statistical methods and was able to generate statistically significantly better results. Another example is the work of Raza where objects in images were successfully classified with the help of a random forest algorithm^30^. In the academic literature, Random Forest is often established and combined as part of an algorithm. The application methods are diverse and are successfully implemented, for example, in gas leak detection^8^, in the detection of plant species^6^ or in maximum power point tracking of solar systems^31^.

#### Fibre volume fraction in different concentration regimes

A significant amount of work quantifies how the fibre volume fraction $\phi$ modifies both macroscopic stresses and orientation dynamics. For Newtonian matrices, classical slender-body theory predicts that the fibre-induced extra stress increases approximately linearly with the volume fraction in the dilute-to-semidilute range, with a prefactor that depends on the aspect ratio (*ar*) and the local orientation state^3,10^. In Jeffery/Folgar–Tucker-type orientation models^14^, hydrodynamic rotary diffusion and direct interactions are often represented by an effective diffusion term whose coefficient increases with the volume fraction $\phi$. However, at $\phi \ll ar^{-1}$ this contribution is small and the kinematics are well captured by the deterministic Jeffery dynamics.

When the matrix exhibits a yield stress (Bingham-type rheology), slender-body/cell-model analyses show that fibres add an anisotropic, orientation-dependent contribution to the stress^13^. This yields a $\phi$-dependent apparent yield stress in the suspension and slightly alters the orientation kinetics by favouring isotropisation in low-shear regions. These effects vanish in the Newtonian fluid and are therefore negligible for the present silicone oil experiments.

## Materials and methods

The liquid being measured is a Newtonian fluid. This liquid contains tiny particles that allow detailed velocities to be measured by visualising them in the flow area using a laser. (See chapter “PIV”.) The quantity of particles is selected so that they do not influence the flow but are sufficient to allow a detailed recording of the flow domain. These are rhodamine B particles made of PMMA.

Fibres are also embedded in the liquid. These are present in small quantities. Due to a similar refractive index as the silicone oil, they are not highly visible so that the PIV measurement is not optically disturbed. See Table 1 for more details of the suspension and the experimental setup.

Five experiments were used to evaluate the fibre orientation; one measurement with one revolution was sufficient to measure the velocity field.

Different evaluation window sizes are compared with each other in the orientation evaluation. In this work, an evaluation window size of 40*40 pixels is chosen. (See chapter "Evaluation of orientation") 5 to 10 fibres can be detected in all evaluation windows in the flow area (total fibre lengths), which ensures a sufficient amount of data per evaluation window.

The python library openpiv is used to analyse the image pairs of the PIV measurements. Here, 32 double images are used to evaluate the velocity field in Fig. 5. The python library sklearn is used for the random forest classifier. The cv2 library is used for preprocessing and other computervision methods. (See chapter “Fibre tracking”)

**Table 1:** Data of the fluid and the experimental setup.

Fluid and components	Symbol	Value	Unit
Viscosity of Silicon oil	$\eta _S$	10,000	$\frac{\text {mm}^2}{\text {s}}$
Particle diameter	$d_P$	0.035	mm
Emitted light of Particles	$\lambda _P$	584	nm
Concentration Particles	$c_P$	0.057	$\frac{\text {g}}{\text {L}}$
Resolution	*R*	6.9	$\frac{\text {pixel}}{\text {mm}}$
Fibre length	$l_F$	30	mm
Fibre diameter	$d_F$	0.16	mm
Single fibre mass	$m_F$	0.8	$10^{-3}$g
Fibre volume fraction	$\phi$	$5.9\times 10^{-3}$	vol.-%

Specifications of the experiment	Symbol	Value	Unit
Angular velocity	$\omega$	0.1	$\frac{\text {1}}{\text {s}}$
Diameter outer cylinder	*D*	240	mm
Diameter interfering body	$d_B$	40	mm
Wavelength of laser	$\lambda$	532	nm

### Fibre volume fraction

In fibre suspensions, the fibre volume fraction $\phi$ and the aspect ratio $ar = l_F/d_F$ govern both hydrodynamic interactions and the likelihood of direct contacts.

The volume fraction is calculated by multiplying the number of fibres *N* by the single fibre volume $V_{\text {fiber}}$ and dividing it by the total liquid volume $V_{\text {liquid}}$:

1 $$\begin{aligned} \phi \;=\; \frac{N \cdot V_{\text {fiber}}}{V_{\text {liquid}}} \;\equiv \; 5.9\times 10^{-3}\,\text {vol.-}\%. \end{aligned}$$

The dilute-to-semidilute crossover for slender rods scales as $\phi ^*\sim ar^{-1}$^3,10,14^. For $ar\approx 188$, $\phi ^*\approx 1/ar \approx 5.3\times 10^{-3}$ (i.e. about 0.53 vol.-%). Our experiments operate almost two orders of magnitude below this crossover: $\phi /\phi ^*\approx 0.011$. This ensures that the hydrodynamic fibre–fibre interactions are weak (dilute regime), the probability of fibre–fibre contact and long-lived clustering is very low, and the fibre-induced extra stresses remain small compared to the matrix stresses.

Practically, the chosen $\phi$ also meets the optical constraints of PIV and fibre detection. It avoids occlusion and preserves a sufficient signal-to-noise ratio. Finally, when $\phi$ approaches or exceeds $\phi ^*\sim ar^{-1}$, contact networks and clustering become increasingly likely^26,38^. In that regime, both the macroscopic rheology (e.g. effective viscosity and apparent yield stress) and the orientation kinetics deviate from the dilute predictions and must be accounted for explicitly (see also Sect. “Scientific fundamentals”).

### Experimental setup

**Fig. 3:**
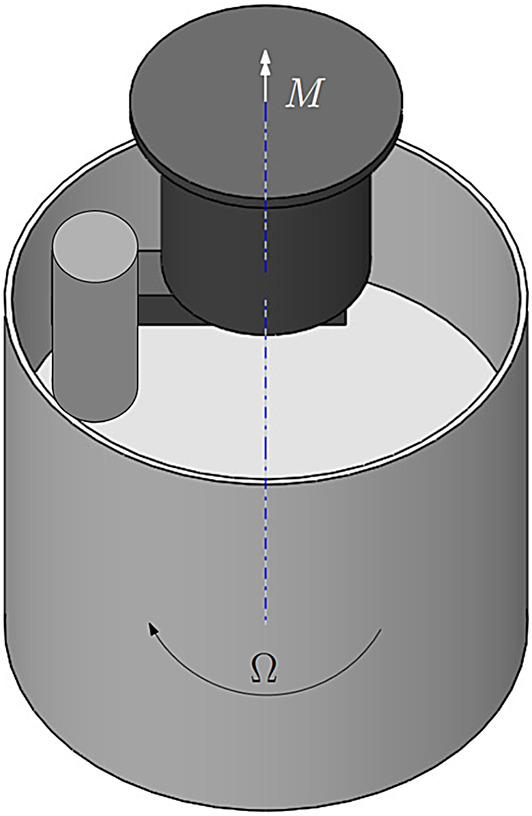
The measuring device used in this paper is based on test systems for viscosity measurement with a cylinder.

This configuration is chosen for the experimental setup in order to be able to relate the influence of a test specimen directly to the fibre orientation. A cylinder is chosen as the test specimen. This allows a flow that is easy to analyse, which has a flow independent of the height and also a fibre orientation independent of the height (plane flow) at a sufficient distance from the floor. This allows the fibre orientation to be examined through the entire measurement configuration, which greatly increases the number of fibres to be examined per measurement. See Fig. 3.

**Fig. 4:**
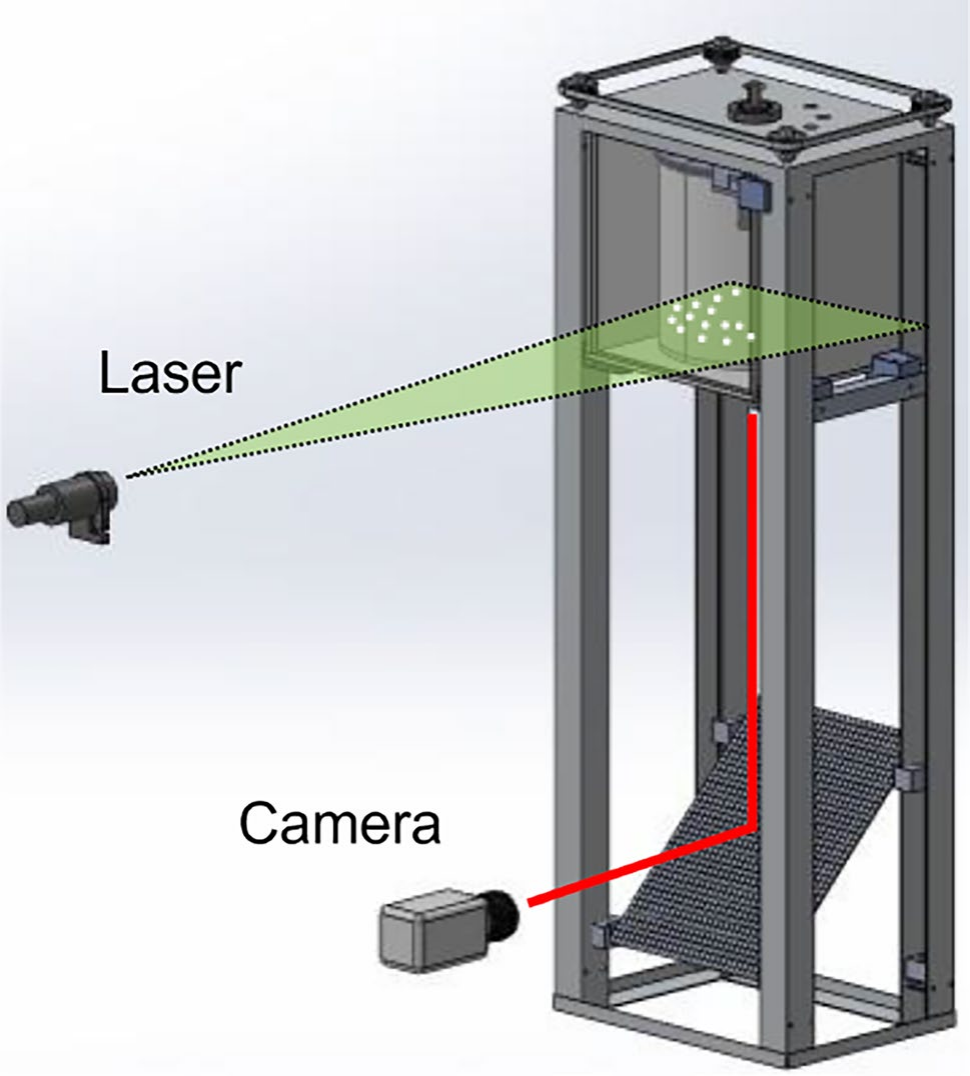
Setup of the measurement with the viewing perspective of the PIV and orientation measurement.

For the PIV measurement, a light section is made at a height of 5 cm from the bottom of the container with a light section thickness of 1.6 mm. The laser for visualisation is arranged as shown in Fig. 4.

The camera observes the flow from below via a mirror. The recording is triggered by the time of exposure of the experiment with the laser. In this set-up the inner cylinder i.e. the interfering body is held in place while the cylindrical container rotates. This allows a stationary measurement of the flow conditions.

### PIV

As described in chapter "Flow visualisation methods", PIV is a common method of flow visualisation to stimulate small particles with a laser and take pictures of them in certain time steps in order to determine a detailed vector velocity field.

**Fig. 5:**
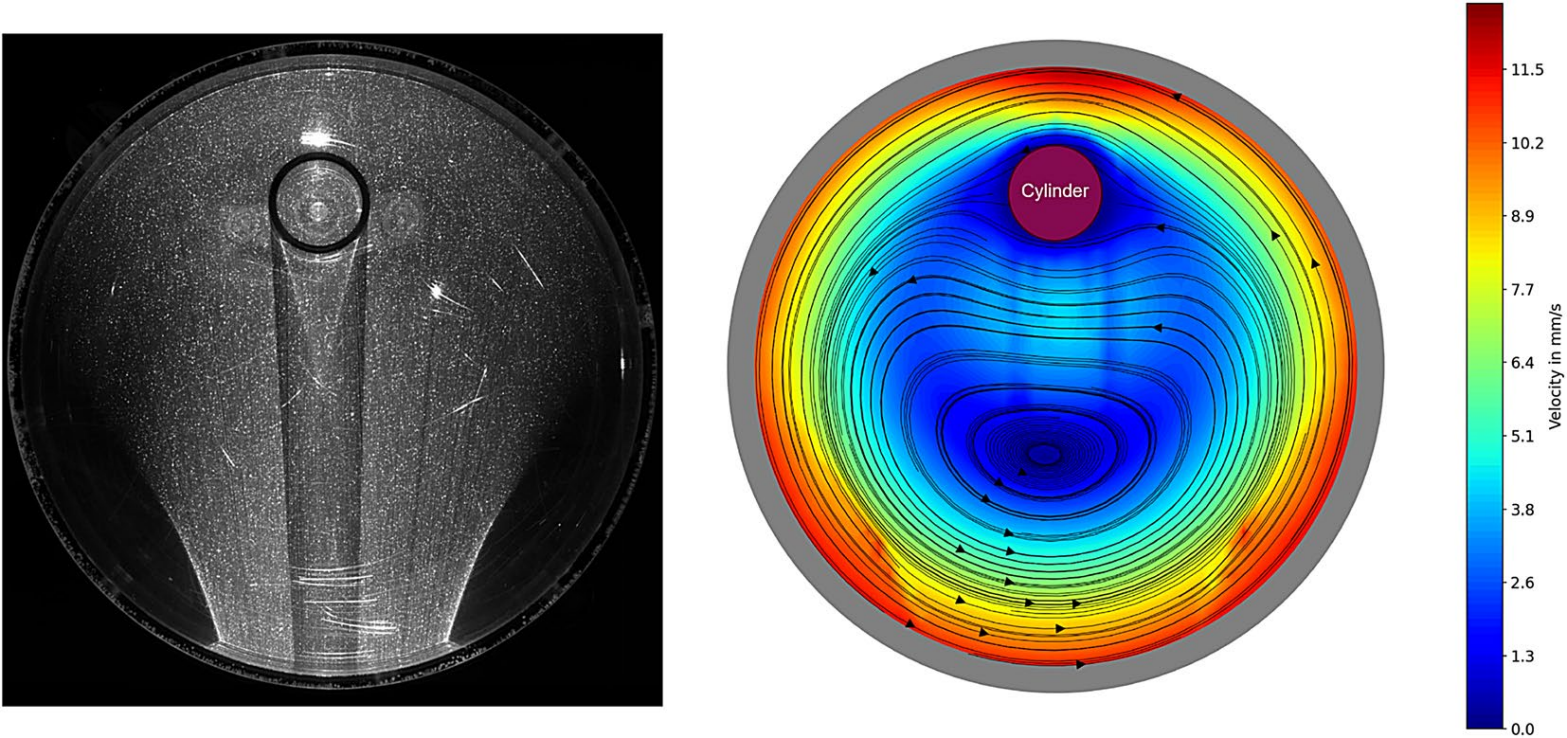
Picture of the Lasercut (left) and the processed Velocity Map with the calculated Streamlines (right).

Figure 5 shows an image of the particles excited by the laser (left). It can be seen that the particles are clearly visible in the laser cut. In addition, individual fibres located in the light section are visible, which allows an initial exemplary analysis of how the fibres behave during the experiment.

Due to the refraction of light at the container and at the interfering body, parts of the image are shadowed. In addition, areas that are overexposed are recognisable. These areas, particularly in the south-western and south-eastern areas of the container, must be explicitly considered when analysing the image. Nevertheless, a detailed velocity field could be calculated by a suitable choice of evaluation windows, which is in good agreement to numerical solutions of this flow.

### Flowtype theory

Figure 5 (right) shows the velocities and streamlines resulting from the PIV measurement. A velocity vector field $\vec {v}(x,y)$ is available from the PIV measurement for each evaluation window. This forms the basis for the calculation of the streamlines in Fig. 5 (right) and for the following determination of the velocity gradient tensor $\textbf{L}$, its symmetrical distortion velocity gradient tensor $\textbf{D}$ and the skew-symmetrical rotational velocity gradient tensor $\textbf{W}$.

In the first step, a velocity gradient field is determined from the velocity vector field $\vec {v}(x,y)$. The central difference quotient is used for this. For instance, the gradient in the x-direction is computed as follows:

2 $$\begin{aligned} \frac{\partial u(x,y)}{\partial x} \approx \frac{u(x,y + \Delta x) - u(x,y - \Delta x)}{2 \Delta x} \end{aligned}$$

Where $\Delta x$ corresponds to the width of an evaluation window. With this the velocity gradient tensor $\textbf{L}(x,y)$ can be formed:

3 $$\begin{aligned} \textbf{L}(x,y) = \begin{bmatrix} \frac{\partial u(x,y)}{\partial x} & \frac{\partial u(x,y)}{\partial y} \\ \frac{\partial v(x,y)}{\partial x} & \frac{\partial v(x,y)}{\partial y} \end{bmatrix} \end{aligned}$$

To further analyse the flow field, the tensor $\textbf{L}$ is broken down into the symmetrical and the skew-symmetrical components:

4 $$\begin{aligned} \textbf{D}= & \frac{1}{2} (\textbf{L} + \textbf{L}^T) \end{aligned}$$

5 $$\begin{aligned} \textbf{W}= & \frac{1}{2} (\textbf{L} - \textbf{L}^T) \end{aligned}$$

The flowtype can be analysed using $\textbf{D}$ and $\textbf{W}$ in the flowtype-function:

6 $$\begin{aligned} f = \frac{\Vert \textbf{D}\Vert - \Vert \textbf{W}\Vert }{\Vert \textbf{D}\Vert + \Vert \textbf{W}\Vert } \end{aligned}$$

where $\Vert \textbf{D}\Vert$ and $\Vert \textbf{W}\Vert$ describe the values (or norms) of the tensors $\textbf{D}$ and $\textbf{W}$, the norm for $\textbf{D}$ being shown here as an example:

7 $$\begin{aligned} \Vert \textbf{D}\Vert = \sqrt{|d_{11}|^2 + |d_{12}|^2 + |d_{21}|^2 + |d_{22}|^2} \end{aligned}$$

where $d_{ij}$ are the elements of $\textbf{D}$.

This results in a value *f*(*x*, *y*) in the range [−1,1] for each evaluation window of the PIV measurement. In the event that the tensor $\textbf{D}$ is the dominant factor, the flow can be classified as planar extensional. Conversely, should tensor $\textbf{W}$ take precedence, the system resides within the negative spectrum of *f*, indicating a rotational flow. Furthermore, when positioned within the range $f (x,y) \approx 0$, the flow is characterized as a simple shear flow:

- $$f = -1:$$ rotational flow
- $f = ~~0:$ simple shear flow
- $f = ~~1:$ planar extensional flow

### Fibre tracking

The following section of this work will focus on measuring and analysing fibre orientation in the flow.

**Fig. 6:**
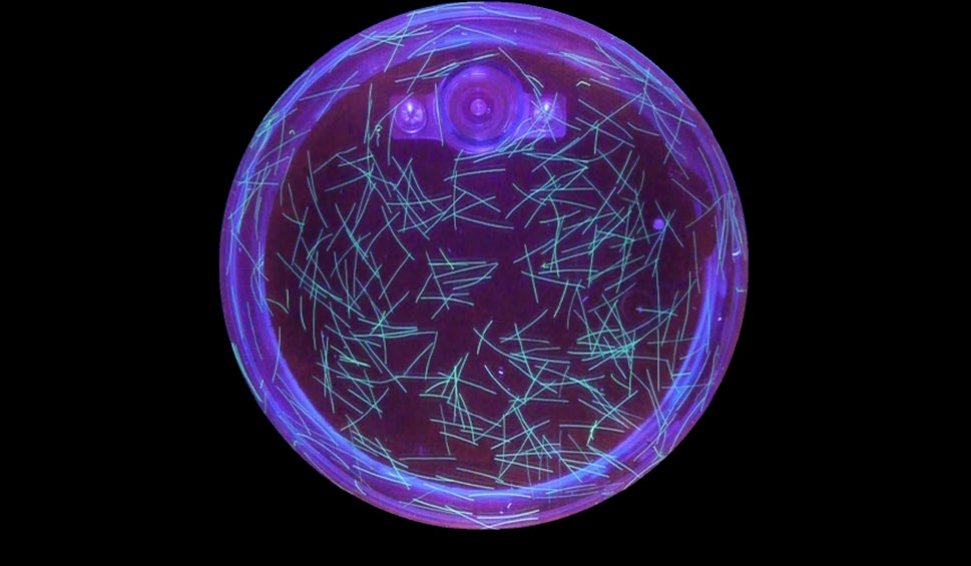
Fibres highlighted by irradiation with black light. View through the entire liquid.

Figure 6 shows an image of the liquid under black light irradiation. A difference between this measurement and the PIV measurement from chapters "Flow visualisation methods" is that images of the entire liquid in the container are taken here in order to have sufficient fibres available for the evaluation of each measurement.

The aim of this measurement is to investigate the actual orientation of individual fibres as well as the mean local orientation. Challenges that need to be considered for measurement:

- Influence of the bottom of the experiment
- Sedimentation of the fibres
- Sufficient randomisation (orientation and volume concentration) in every area at the beginning of the experiment
- Bubble creation when mixing the liquid
- Sufficient repetitions of the experiment

Based on these requirements, fibres are selected with a density similar to that of the matrix liquid to minimize sedimentation effects. Although a perfect density match cannot be achieved, the resulting buoyant force becomes negligible due to the high viscosity of the liquid. To prevent bubbles from interfering with the measurement, the liquid should be allowed to rest for at least 12 hours after mixing, enabling trapped air to dissipate before any measurements are taken.

Sufficient randomisation of the fibres at the start of the experiment was achieved in most parts of the container. However, near the container walls and in the immediate vicinity of the interfering body, random orientation of the fibres is not possible. This is due to wall-parallel flow generated during mixing in these regions, which forces the fibres to align along the wall rather than assume random directions. In addition, fibre concentration near the wall is reduced due to wall–fibre interactions.

**Fig. 7:**
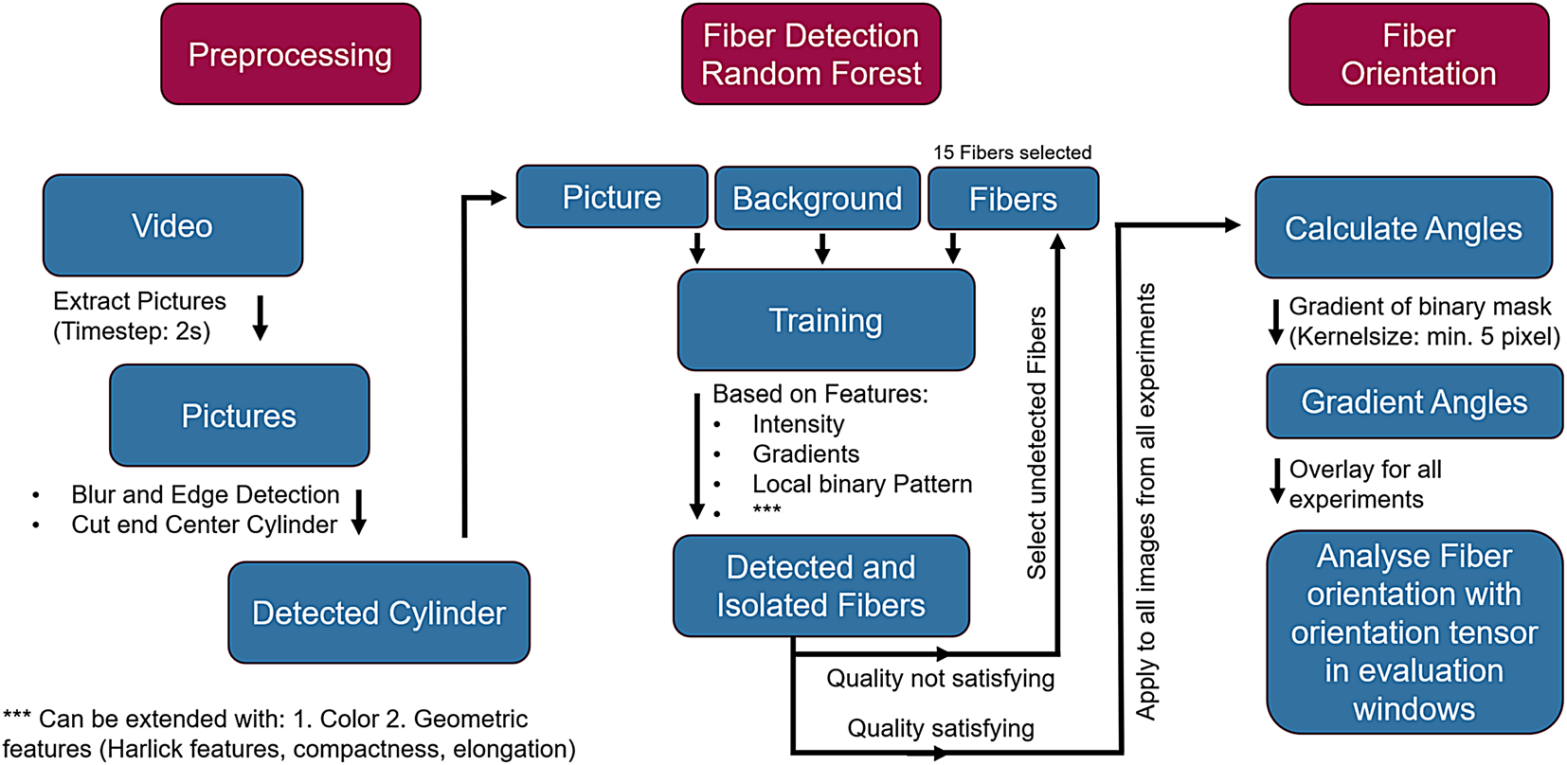
Process diagram of fibre detection with the Random Forest algorithm, preprocessing and fibre orientation calculation.

Since the measurement captures the fibre orientation across the entire fluid volume, fibres near the bottom of the container also contribute to the result. Their influence can distort the measurement if the bottom region differs significantly from the bulk. This effect is mitigated by ensuring a sufficient fill height and by applying targeted evaluation methods that focus on representative regions. To ensure statistical reliability, five experiments are conducted and analysed. The procedure for analysing the recorded video of the fibre orientation measurement is shown in Fig. 7.

The video is divided into a sufficient number of images to minimise the computing effort. In addition, all elements outside the experiment (i.e. outside of the flow domain) are masked. The time step must be large enough so that a sufficient change to the previous image can be seen in a subsequent image, but the fibres have moved as far as maximum to the adjacent evaluation window. An optimum of low calculation time and sufficient time resolution is found with a time step of 2 seconds.

In the following, a random forest algorithm is used to detect the fibres on the respective images. These fibres are then used to perform an orientation analysis. (See also chapter "Analysis of the orientation")

#### Randon forest classifier

In order to be able to use the fibres on the images for orientation analysis, they must be tracked and separated from the background. The following section deals with tracking these fibres with a Random Forest AI Classifier with the basic structure of which is described in chapter "Fibretracking with random forest".

As the training only takes a very short time, many learning phases could be analysed and compared. The Random Forest was initially trained with a few fibres and the results visually evaluated. As this is a classification task, the algorithm searches for fibres similar to those in the training data set. It could be seen that certain types of fibres are not or insufficiently recognised. Additional fibres are therefore successively selected whose type (short fibres, poorly exposed fibres, differently exposed fibres, strongly curved fibres, superimposed fibres,...) are not recognised well. After several iterations, 15 fibres were found that could cover all fibre types and give a satisfactory result. (See Fig. 7)

It is also important for the random forest algorithm to select where the background is located. This defines a further class that provides a clear differentiation between the fibres and the background. This can also be done successively and if the background is incorrectly recognised as a fibre, the background can be extended until a clear separation is achieved.

Another important decision to find the fibres reliably is which properties are taken from the images and made available to the algorithm. (See also chapter "Fibretracking with random forest") It is sufficient to use the intensity, the gradient to surrounding pixels (5x5 pixels) and the local binary pattern (which describes a detailed view of the surrounding pixels) for the images taken. Using additional properties did not achieve any significant improvements, but greatly increased the computational effort.

**Fig. 8:**
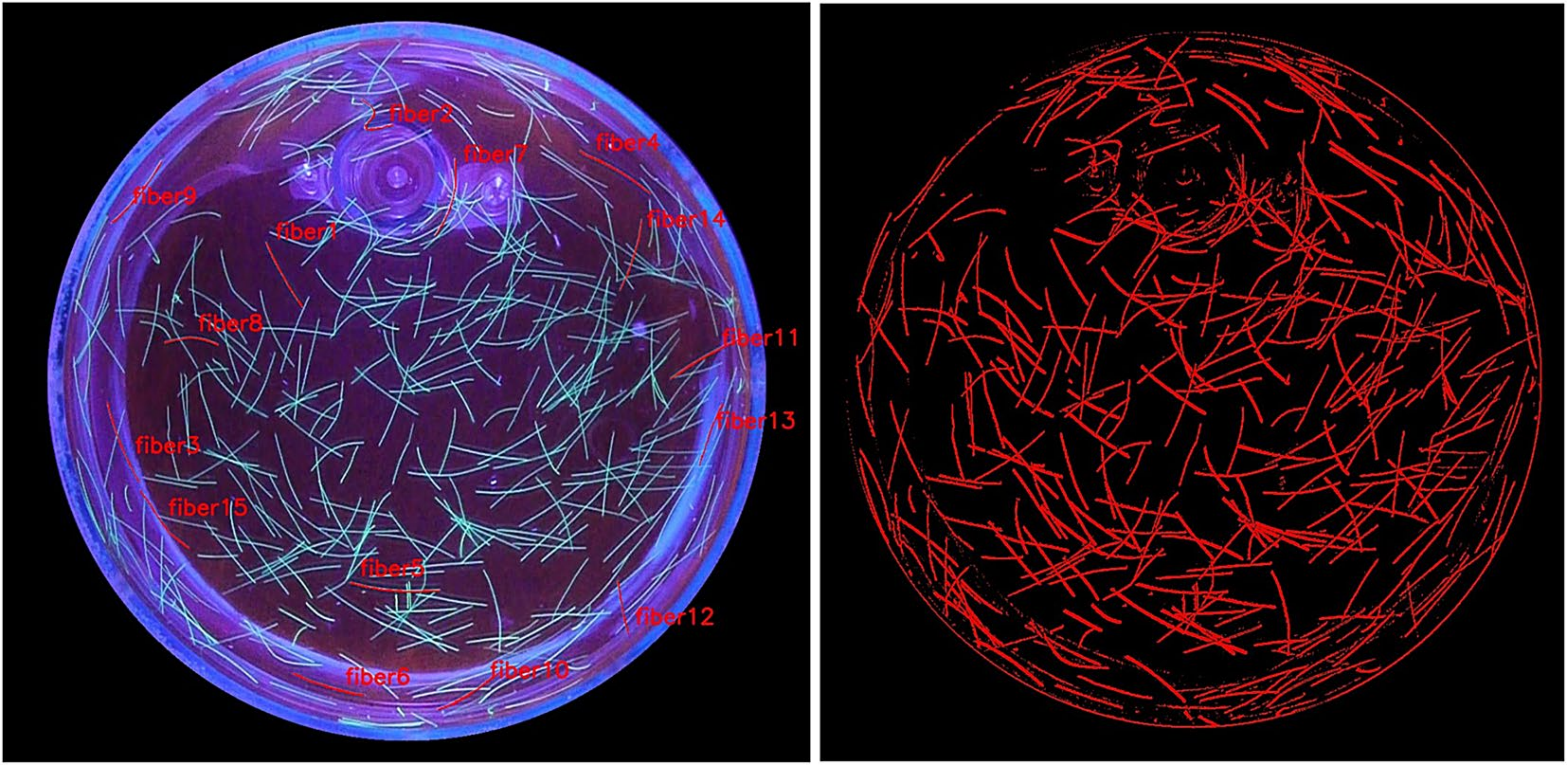
Fibres used for Training the Random Forest algorithm (left) and the result of fibre detection for the training image (right).

It can be shown in Fig. 8 that with a training data set of 15 selected fibres, all other fibres can be found on the training image. In addition, all fibres can be reliably found on all other images of the experiment and all other experiments.

## Results and discussion

### Evaluation of the flow

#### Reynolds number

To check which forces lead to this flow, the Reynolds number is calculated. The Reynolds number using the diameter of the cylinder with a maximum velocity of $U_{max} = {12.4}\hbox {mm}\,\hbox {s}^{-1}$ results in:

8 $$\begin{aligned} Re_{max} = \frac{d~U_{max}}{\nu } = 5 \times 10^{-2} \end{aligned}$$

The Reynolds number is therefore $\ll 1$, which means that the frictional forces predominate and the inertial forces are negligible, resulting in a creeping flow. It can therefore be argued that the speed of rotation of the container has a negligible influence on the flow and therefore has no influence on the trajectories or fibre orientation. Experiments were carried out with higher and lower angular velocities, which confirm this statement, as long as the Reynolds number is sufficiently small.

#### Determination of strain and shear rates

**Fig. 9:**
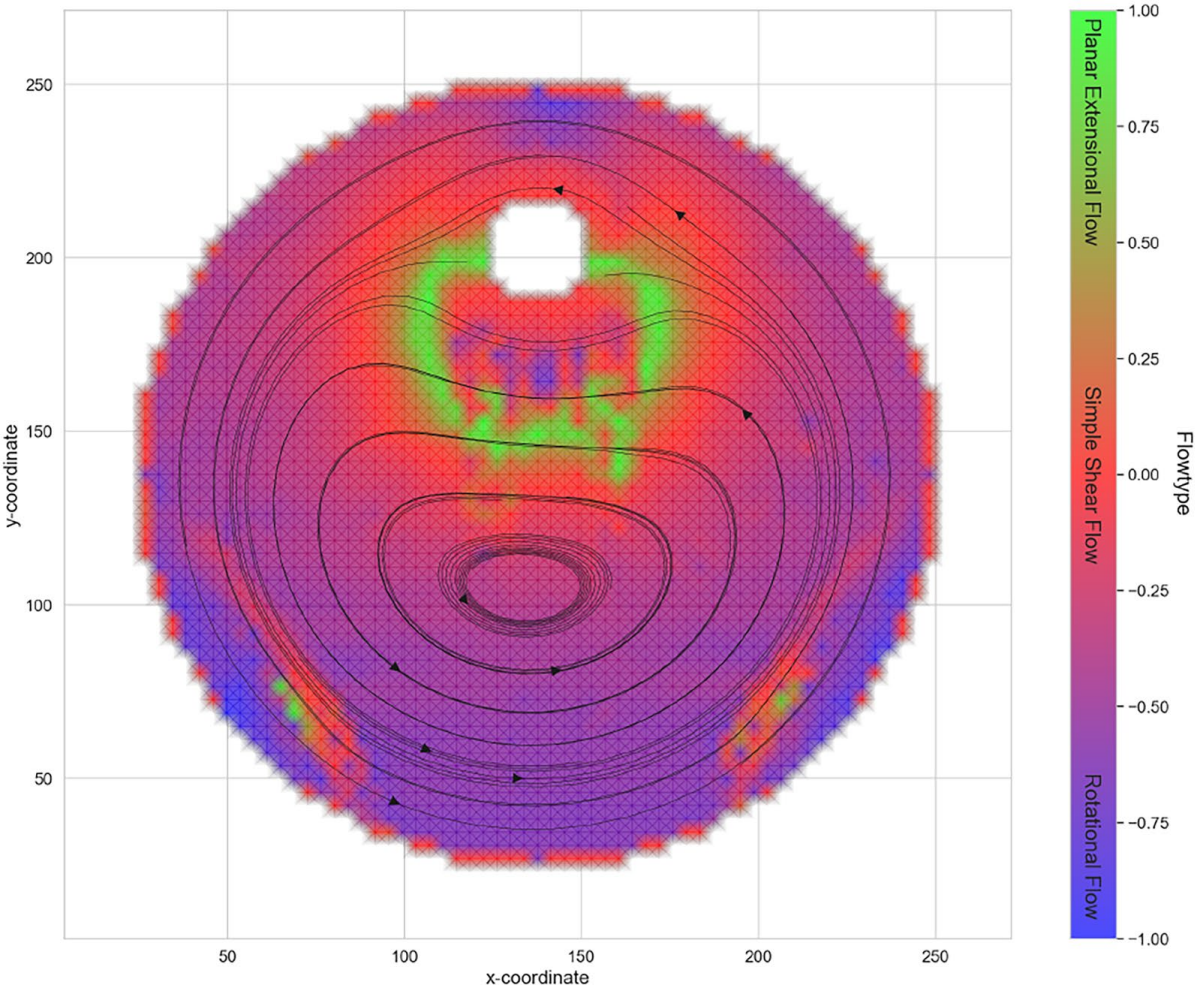
Visualisation of the flowtype in the flow area.

In this chapter, the theory shown in chapter “Flowtype theory” is now used and applied to the present flow area. In the current observations, no constant flowtype can be distinctly identified; rather, we primarily encounter mixed regions, as illustrated in Fig. 9 for the complete flow region.

Figure 9 shows that all characteristics of the value range of the flowtype *f* can be found in the measuring range. A dominant rotational flow can be observed in the southern region, far from the flow around the cylinder. The closer to the cylinder, the more shear flow appears. An extensional flow can be seen in a circular shape below the interfering body. The respective flow conditions are responsible for how fibres that are transported with the flow orient themselves. Fibres are oriented in the direction of the streamlines in shear^25,34,35^. In a rotational flow, however, the fibres do not orientate themselves. In an extensional flow, fibres are oriented either in the direction of the flow (elongation) or perpendicular to the flow (compression)^39^. For this reason, the direction and value of the strain rate and shear rate must be determined for a more precise analysis. This can be done by bringing the tensor $\textbf{D}$ to main axis shape, whereby the eigenvalue problem must be solved, from which the two eigenvalues emerge and can be used to form the tensor $\mathbf {\widetilde{D}}$ as follows:

9 $$\begin{aligned} \mathbf {\widetilde{D}} = \begin{bmatrix} \lambda _{D1} & 0 \\ 0 & \lambda _{D2} \end{bmatrix} \end{aligned}$$

with the eigenvalues $\lambda _{D1}$ and $\lambda _{D2}$, where $\lambda _{D1}$ is defined as the smaller eigenvalue. In addition, it should be noted that the sum of the eigenvalues must be zero for an incompressible flow:

10 $$\begin{aligned} I_D = \text {tr}(\textbf{D}) = \text {tr}(\mathbf {\widetilde{D}}) = \text {div}(\vec {v}) \simeq 0 \end{aligned}$$

This results on the one hand in an error measure, which is analysed in more detail in chapter "Error analysis in PIV", and on the other hand that the magnitude of the eigenvalues is the same:

11 $$\begin{aligned} |\lambda _{D1}|\simeq |\lambda _{D2}|\end{aligned}$$

The respective eigenvalues can be interpreted as strain (elongation or compression) $\dot{\varepsilon }$ or shear $\frac{\dot{\gamma }}{2}$ depending on the calculated flowtype *f* at the respective position:

12 $$\begin{aligned} \mathbf {\widetilde{D}} = \begin{bmatrix} \lambda _{D1} & 0 \\ 0 & \lambda _{D2} \end{bmatrix} ~~~=~~~ \begin{bmatrix} -\dot{\varepsilon } & 0 \\ 0 & \dot{\varepsilon } \end{bmatrix} ~\text {or}~ \begin{bmatrix} -\frac{\dot{\gamma }}{2} & 0 \\ 0 & \frac{\dot{\gamma }}{2} \end{bmatrix} \end{aligned}$$

#### Direction of the extensional flow

As the orientation of the fibres depends on the type of strain flow (elongation or compression), a further analysis of the strain direction must be carried out. To do so, the eigenvectors $\vec {k}_{D1}$ and $\vec {k}_{D2}$ of the tensor $\textbf{D}$ are multiplied by the present velocity vector $\vec {v}$ to determine the sign of the strain flow, which is then multiplied by the magnitude of the eigenvalue $\lambda _{D1}$ or $\lambda _{D2}$:

13 $$\begin{aligned} \dot{\varepsilon } = {\left\{ \begin{array}{ll} - |\lambda _{D1} |~~~~~\text {if}~~~~~ |\vec {k}_{D1}\cdot \vec {v}|> |\vec {k}_{D2}\cdot \vec {v}|\\ + |\lambda _{D1} |~~~~~\text {if}~~~~~ |\vec {k}_{D1}\cdot \vec {v}|< |\vec {k}_{D2}\cdot \vec {v}|\\ \end{array}\right. } \end{aligned}$$

The larger scalar product indicates which of the eigenvectors $\vec {k}_{D1}$ or $\vec {k}_{D2}$ is oriented closer to the velocity vector. If the magnitude of the scalar product with the first eigenvector $\vec {k}_{D1}$ is greater, the sign of the associated first eigenvalue $\lambda _{D1}$ (In two-dimensional space, always negative by definition) defines the direction of the strain, is negative in this case and thus describes a compression. This can be shown for elongation if the scalar product with the second eigenvector $\vec {k}_{D2}$ predominates in terms of magnitude.

**Fig. 10:**
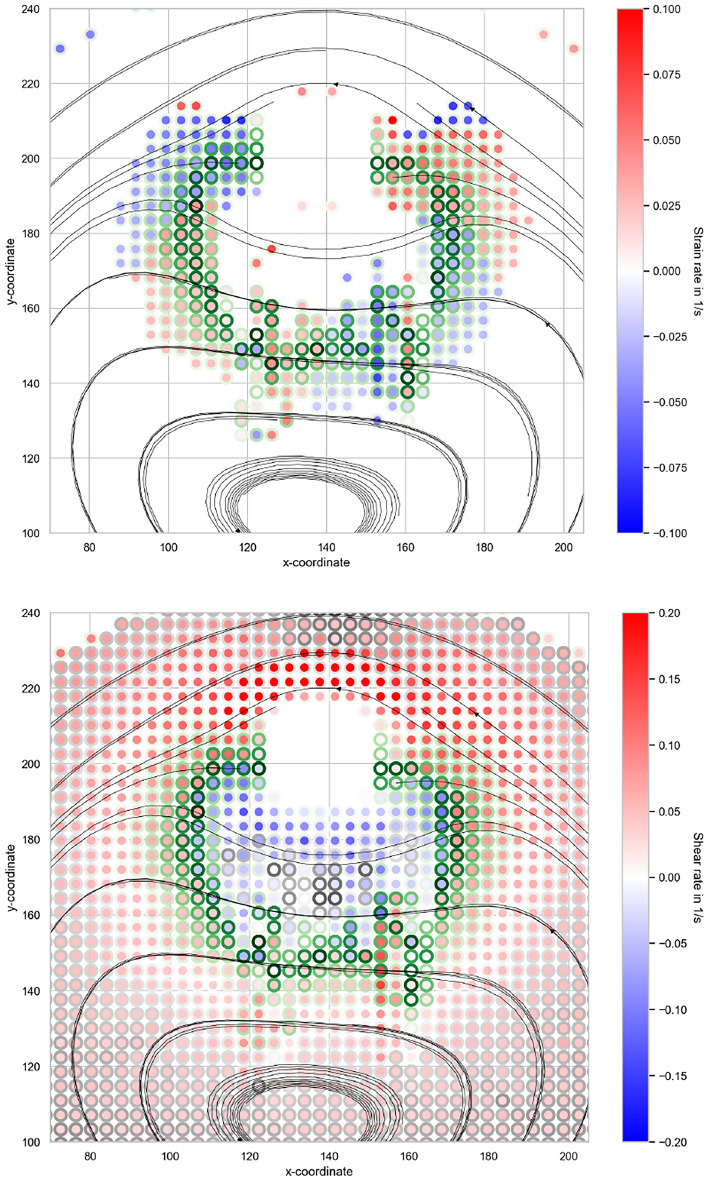
Representation of the strain rate in the flowtype area $f> 0$ (top) and the shear rate in the entire flowtype area (down). The border of the circles representing the flowtype *f* value with the colour scheme for the extensional flow in green as used in Fig. 9, a white edge for a shear flow and a grey edge for a rotational flow.

Figure 10 (top) shows the strain rate in the areas with flow type $$ f > 0$$, where a strain flow (at least partially) is present. It can be seen that there is compression of the area in front of the interference body. In comparison, there is an elongation of the flow after the interfering body. To the south-east of the interfering body there is also an elongation and to the south-west there is a compression. Figure 10 (down) shows the shear rate. It can be seen that the strain flow under the interfering body shows a kind of separating circle where the direction of the shear differs. The greatest shear occurs above the disturbing body and the further you are from the disturbing body, the lower the shear.

#### Error analysis in PIV

The first invariant from equation 10 is zero in theory for an incompressible flow. However, the measurement data shows that errors occur here. $I_D$ should now be shown in relation to the mean eigenvalue of the tensor $\textbf{D}$, which defines the error as follows:

14 $$\begin{aligned} I_{D;error} ~~=~~ I_D : \frac{1}{2} \sum _{n=1}^2 \lambda _{Di} ~~=~~ \frac{\lambda _{D1} + \lambda _{D2}}{\frac{1}{2}(|\lambda _{D1}|+ |\lambda _{D2}|)} \end{aligned}$$

**Fig. 11:**
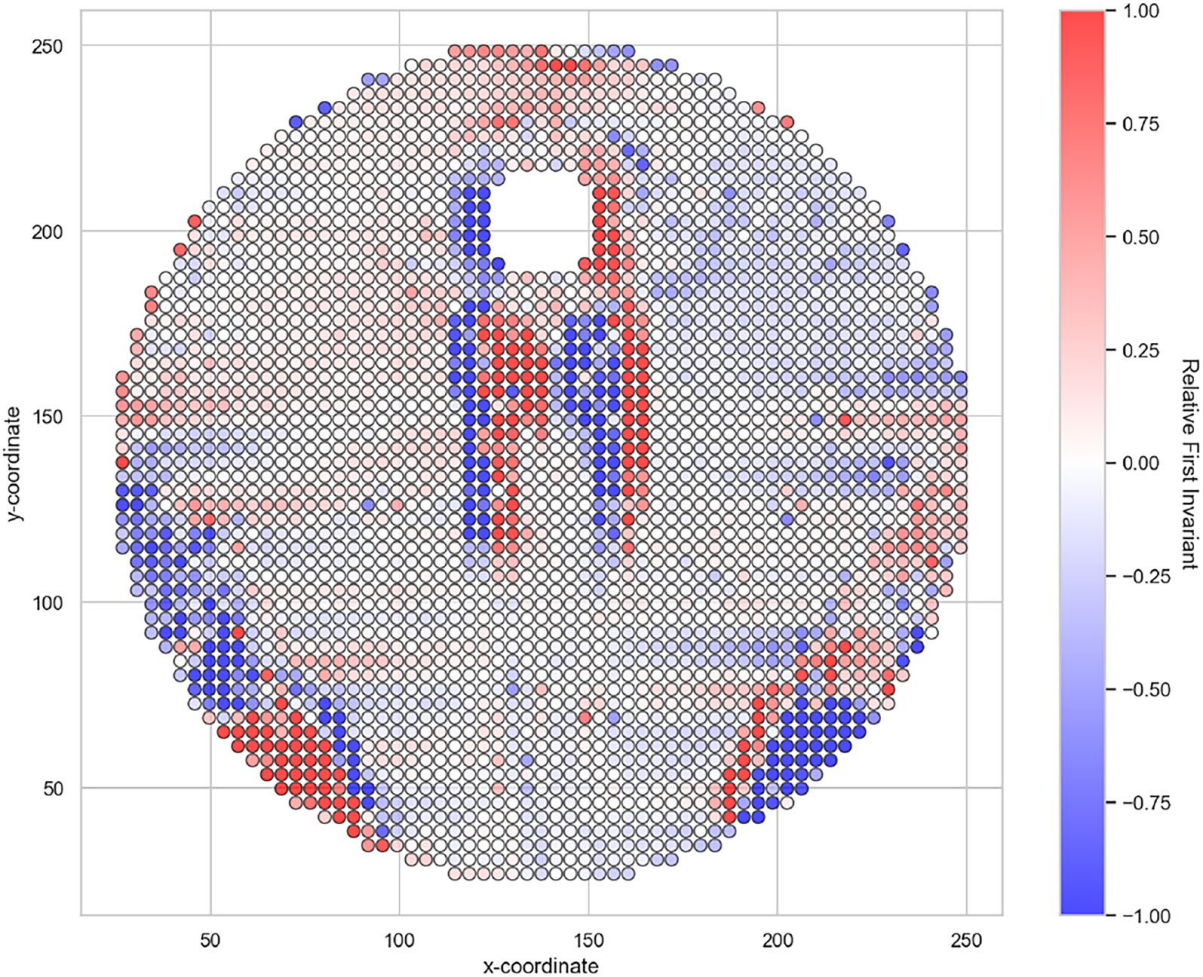
Relative error of the first invariant $I_D$ with respect to the mean value of the eigenvalues of $\textbf{D}$.

The error is displayed for the whole experiment in Fig. 11. Significant errors can be seen in the areas south the interfering body. As can be seen in Fig. 5 (left), shadow areas can be recognised there due to the refraction of light. These errors can also be seen in the areas to the south-west and south-east of the cylinder due to the presence of shadow areas. These errors should be taken into account when assessing the results in these areas. As the error value is formed with the eigenvalues, the differences can be recognised in particular in Figs 9 and 10. In the area to the south of the interference body, it can be seen that the extensional flow occurs in a circular shape and is slightly blurred in the fault areas.

It can also be shown that three-dimensionality plays a subordinate role. It can be seen that the flow builds up in front of the disturbance body and recedes to a small level behind the disturbance body, which explains the error directly at the disturbance body in these areas. A small error can be seen in the other areas of the measuring range.

Despite the error due to the shadow areas, a qualitative statement can be made here about the flow types, shears and compressions. The comparison with numerical simulations also shows good agreement with the PIV measurement. A more precise measurement is therefore not necessary for this work. Nevertheless, the exposure should be improved in subsequent work in order to carry out more precise measurements with regard to boundary effects. See chapter “Outlook”.

#### Interim conclusion of flow analysis based on PIV

At this point, the evaluation of the flow region can be concluded on the basis of the PIV measurement.

It turns out that a velocity vector field can be generated from the PIV images, which can be further analysed with regard to shear and strain. As described in the chapter "Determination of strain and shear rate", this shear and strain causes an orienting or deorienting effect on the fibres. In the case of the strain flow, an alternating orienting and deorienting effect can be predicted south of the disturbing body where strain dominates. So, at this point in the evaluation, no precise statement can be made as to how the fibres orient themselves in the southern flow around the interfering body due to the strain flow. In contrast, shear always leads to an orienting effect with regard to the streamlines, regardless of the direction of the shear. It can be determined that a large shear occurs in the vicinity of the interfering body. Only small shears occur far away from the interfering body (southern area of the cylinder), which therefore show a small orientating effect in these areas.

However, the other direction of shear to the south of the obstacle can slow down the orientation of fibres that are not yet oriented. This is because the direction of orientation will also change briefly with the other sign, and thus the orientation will be briefly opposite to the ’faster’ direction of orientation. For fibres that orientated themselves in the direction of the streamlines, the change of direction no longer has any recognisable influence. This is because the orientation due to shear is strongly dependent on the current orientation. It is highest when the fibre is perpendicular to the flow and lowest when the fibres are oriented parallel to the streamlines.

### Analysis of the orientation

After isolating the fibres from the background in chapter “Fibre tracking”, the orientation of the fibres will be analysed in this chapter. At this state of the work, we do not have any individual fibres isolated from each other; we only know which pixels belong to the fibres group and which pixels belong to the background group. We therefore have a so-called binary picture. We could process this image further and separate the fibres from each other. However, we choose a more efficient and detailed approach: the binary gradient, which allows us to calculate every single orientation of every single fibre pixel. If, for example, a fibre lies partly in the direction of flow and partly vertically to it due to its flexibility, a detailed statement of the orientation of each fibre component can be determined here.

A window with the size 7x7 pixels is analysed around each pixel. A gradient is calculated by using the surrounding pixels. For each pixel, the Sobel operator calculates a weighted sum of the pixel values in the area. Essentially, the Sobel operator provides a simple but effective method for edge detection by utilising gradient information derived from convolution with specific direction kernels. This produces a gradient vector that represents the rate of change of the binary values at this pixel position both horizontally ($\text {grad}_x$) and vertically ($\text {grad}_y$). After calculating the gradients, the angle can be calculated using the following function:

15 $$\begin{aligned} \phi = \text {arctan2}(\text {grad}_y, \text {grad}_x) \end{aligned}$$

It is often used with intensity values on images to show differences in non-binary images to neighbouring pixels. The larger kernel size applies the weighting more smoothly than stricter Sobel derivatives, often blending and emphasising transitions across wider regions. We take advantage of this effect in the binary image to calculate clear gradients across fibre boundaries.

**Fig. 12:**
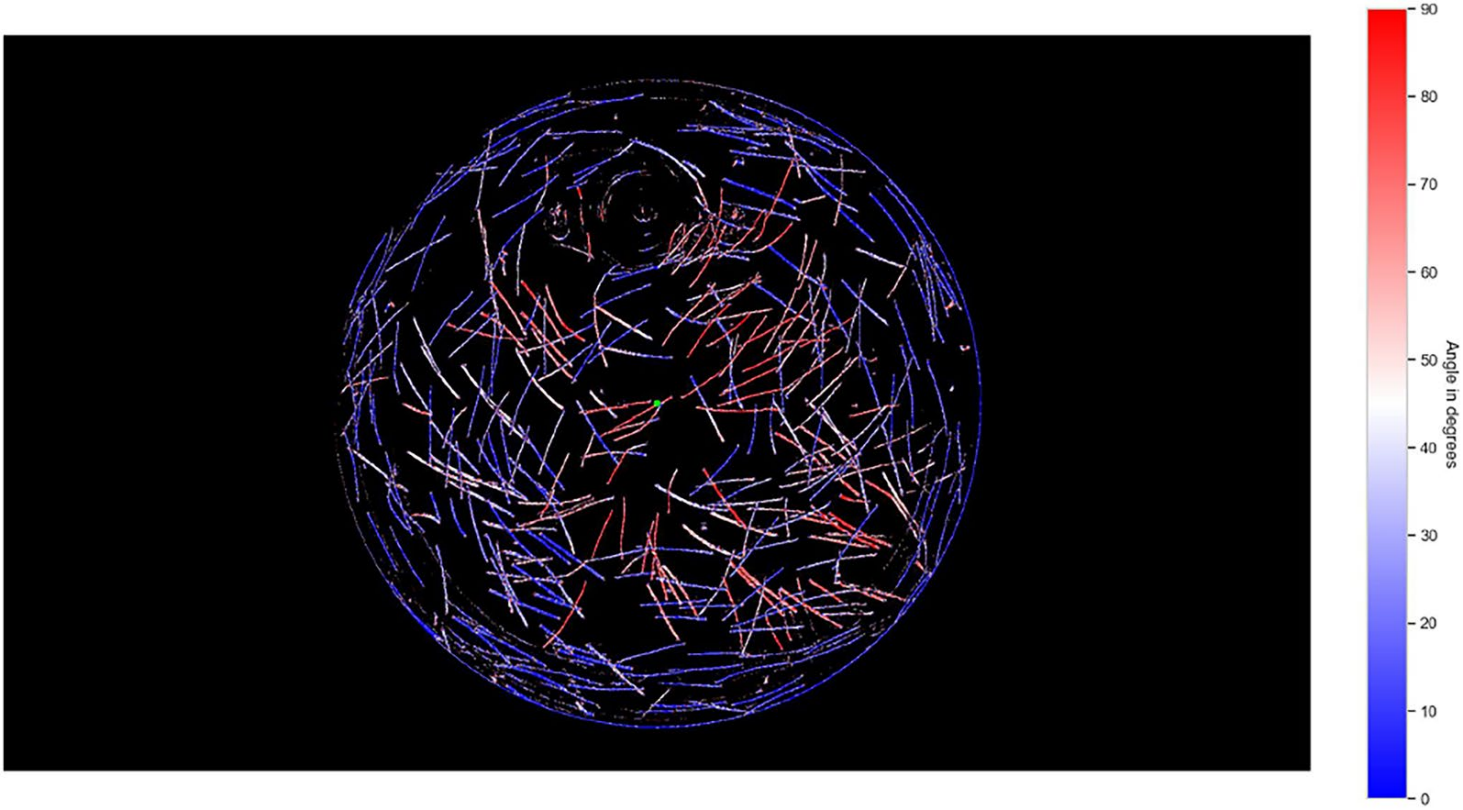
Evaluated fibre orientation for all tracked fibres in relation to the centre of the image or in the circumferential direction. Each section of the fibres is analysed explicitly per pixel.

Figure 12 shows the experiment at a given point with the evaluation of the respective orientation of the fibre pixels using the binary gradient method. It shows the orientation of the pixels of the fibres relative to the centre of the experiment and thus also relative to the cylinder shape. This results in a description of the orientation in the range $[0^{\circ }, 90^{\circ }]$ respectively in the range: [vertically to the centre, pointing towards the centre]. This evaluation is carried out for all generated time steps and for all experiments. This forms the basis for analysing the orientation of the fibres during the measurements.

#### Evaluation of orientation

In order to obtain a more precise analysis of the orientation, the orientations for the entire flow area will be analysed in this chapter. In order to obtain statistically reliable statements, a sufficient amount of data must be available, which ultimately correlates with a low standard deviation.

However, the number of measuring points per experiment is physically limited: on the one hand in terms of time, because the orientation is time-dependent, and on the other hand in terms of location, because the fibre volume fraction as a physical property of the suspension determines how many fibres can be observed in one place and at one time. For these reasons, a sufficient statistical distribution cannot be determined from a single experiment. Assuming that the experiments are reproducible, it can be assumed that the flow process (and thus the fibre orientation) is reproducible at the same time after the start of each experiment (and under the same initial conditions). The random fluctuations within each experiment lead to the statistical distribution of the process. Five experiments are used, which are superimposed at any place and at any time.

This approach ensures that incorrect orientations are reliably excluded. When considering a single fibre in isolation, it is not possible to determine whether its orientation is representative or simply an outlier. However, by superimposing multiple fibres or experimental results, patterns become discernible: if the fibres exhibit varying orientations, this indicates a lack of directional consistency. Conversely, if all fibres consistently align in the same direction across multiple experiments, the orientation can be confirmed with statistical significance – and even the probability distribution over possible orientations can be inferred.

The orientation tensor of Advani and Tucker is used for evaluation^2^, which use the respective orientations of fibres and determine the second-level orientation tensor $\mathbf {A_2}$ in certain evaluation windows. In this experimental approach, instead of using the orientation of entire fibres, the orientation of the respective fibre pixels is used, so that the evaluation windows can also use parts of fibres and also have the advantage, as described in chapter "Analysis of the orientation", that the exact orientation of the fibre part is described. The orientation tensor $\mathbf {A_2}$ is calculated as follows:

16 $$\begin{aligned} \mathbf {A_2}(x,y) = \frac{1}{N} \sum _{i=1}^{N} \vec {p}_i \vec {p}_i ~~~~~~~~~ \text {with:}~~\vec {p}_i = \begin{bmatrix} \cos (\phi ) \\ \sin (\phi ) \end{bmatrix} \end{aligned}$$

Where the respective orientation vectors per pixel are $\vec {p}_i$ and the number of pixels in the respective evaluation window is *N*. It requires an evaluation of the tensor $\mathbf {A_2}$ with respect to the eigenvalues $\lambda _{A1}$ and $\lambda _{A2}$ and the eigenvectors $\vec {k}_{A1}$ and $\vec {k}_{A2}$. To visualise the orientation, an ellipsoid can be spanned with the eigenvectors. The associated eigenvalues indicate the length of the eigenvectors. In two dimensions, the orientation tensor is a 2x2 matrix and therefore has two eigenvalues and two eigenvectors. The sum of the eigenvalues is defined by the first invariant:

17 $$\begin{aligned} {I_A} = sp\{\mathbf {A_2}\} = \lambda _{A1} + \lambda _{A2} \equiv 1 \end{aligned}$$

The representation of the largest eigenvalue $\lambda _{A1}$ is therefore sufficient for the evaluation (in two dimensions), as the second-level eigenvalue $\lambda _{A2}$ is thus fixed and provides no further information. If $\lambda _{A1} = 1$, the analysed area exhibits a perfectly uniaxial orientation. In contrast, if $\lambda _{A1} = 0.5$, the fibre orientation is completely isotropic, indicating no preferred direction. The eigenvector corresponding to the largest eigenvalue indicates the dominant orientation direction of the fibres at that location.

**Fig. 13:**
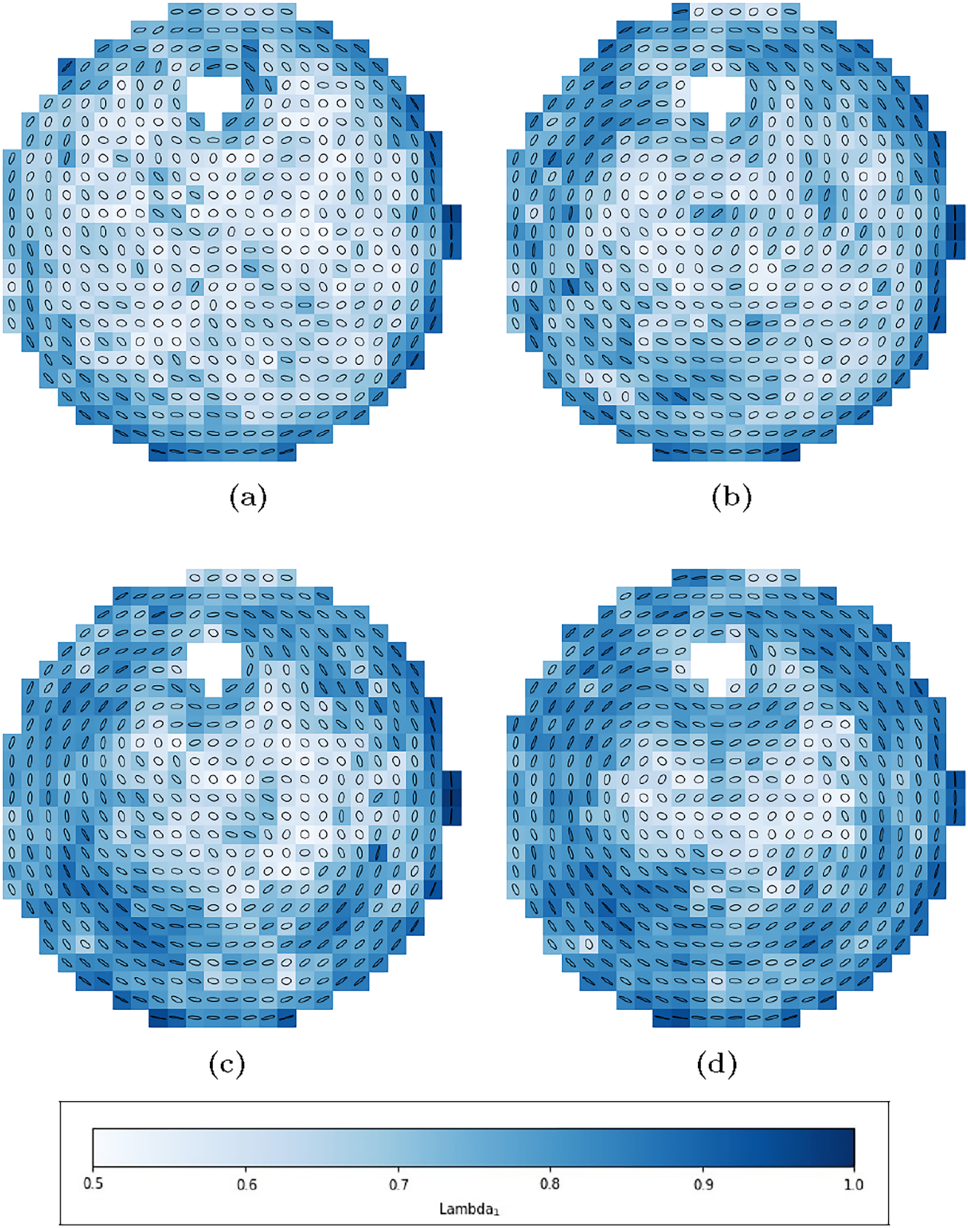
Representation of the orientation of the fibres in the flow area with the respective eigenvectors $\vec {k}_{A1}$, $\vec {k}_{A2}$ and eigenvalues $\lambda _{A1}$, $\lambda _{A2}$ as ellipsoids in the respective evaluation windows at certain times of the measurement.

Figure 13 shows evaluations of the fibre orientation using the ellipsoids during the experiments. Evaluation Windows of 40x40 pixels are used here, which allows us to achieve a sufficient amount of data per evaluation window. (See chapter "Materials and methods")

A rapid and clearly recognisable fibre orientation is observed in certain regions. Fibres situated close to the flow around the cylinder, or carried along with it during the experiment, tend to align with the local streamlines. In the central part of the container, the fibres show either no orientation or only a very weak alignment. These results are consistent with the analysis of the flow field, which shows a strong orientation effect due to shearing in the vicinity of the disturbance body (See chapter "Determination of strain and shear rates" and "Direction of the extensional flow"). It can also be seen that the orientation mainly occurs on the streamlines that flow north and very close to the south of the disturbance body. This confirms the alternating orienting and de-orienting effect under the disturbance body, which slows down the orientation there.

**Fig. 14:**
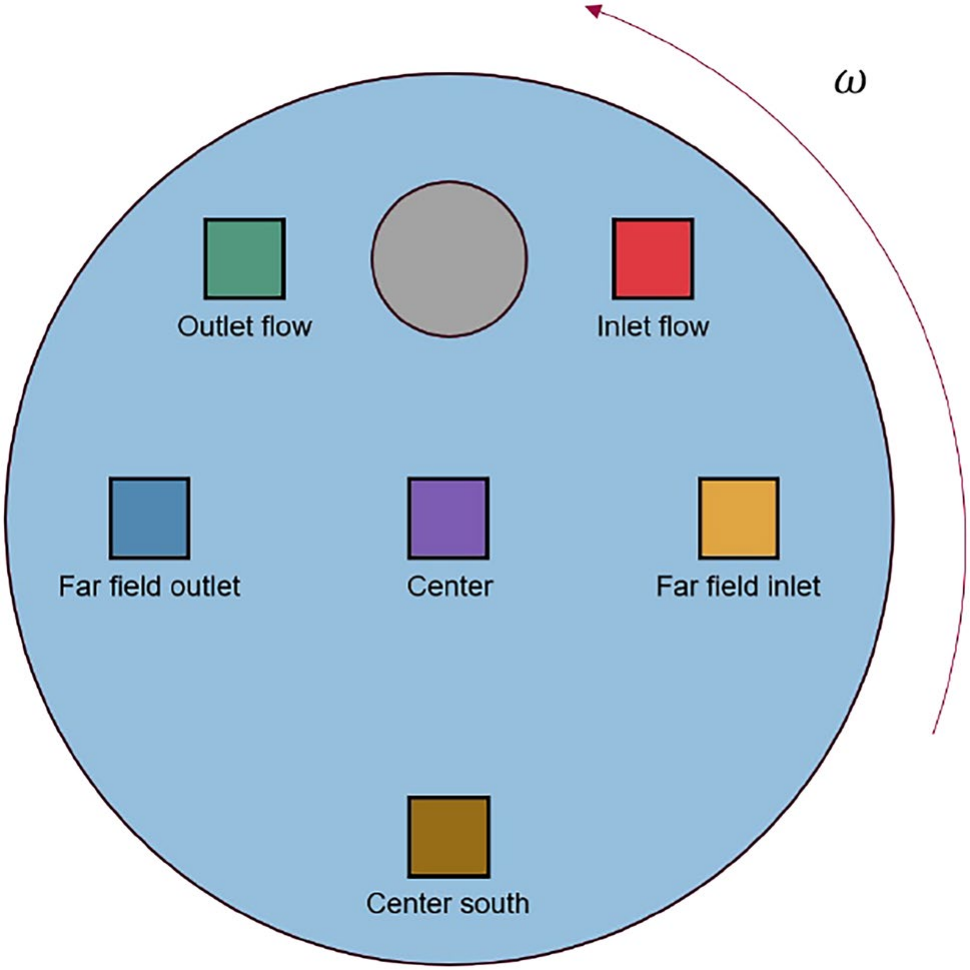
Selected areas for the evaluation of orientation over time from Fig. 15. An evaluation window of 80x80 pixels is used here.

In simulations of fibre orientation, perfectly random orientation is commonly assumed at the beginning of the experiment. The experiment shows that this is not realistic. Due to fibre migration and contact with the wall, explained in chapter “Fibre tracking”, fewer fibres are present here and a pre-orientation is recognised. This can be seen particularly at the edge and in the area between the interfering body and the outer cylinder (Fig. 14).

#### Orientation over time

**Fig. 15:**
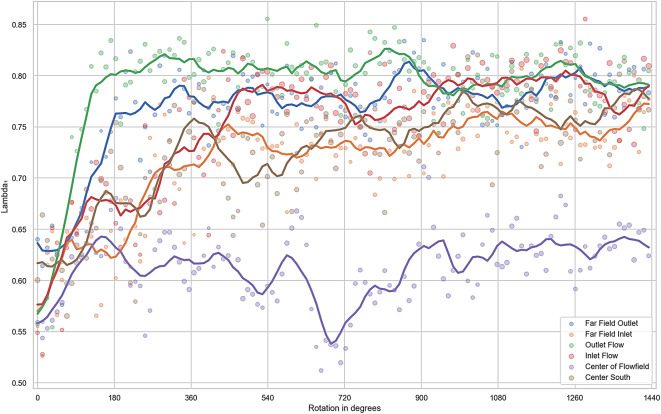
Representation of the largest eigenvalue of the orientation tensor at selected points over time. The dots show the data points used at the respective points in time (5 superimposed experiments in one evaluation point). For this purpose, a centred mean value is displayed over a range of 8 data points.

To gain a more detailed understanding of the orientation process at specific locations, Fig. 15 presents the orientation at selected points, based on the largest eigenvalue of $\mathbf {A_2}$. The corresponding point locations are marked in Fig. 14, and the resulting orientation values are shown in Fig. 15.

Regions of particular interest are selected for this purpose. For example, regions behind and in front of the interfering body, regions far away from the interfering body and a region that shows nearly isotropic orientation according to Fig. 13 are selected.

Figure 15 shows that after half a rotation ($180^{\circ }$), a strong orientation occurs in the ‘Outlet flow’ and ‘Far field outlet’ domains, which stabilises between the values 0.75 and 0.85 as the experiment progresses. In the ‘Centre south’, ‘Far field inlet’ and ‘Inlet flow’ areas, orientation is achieved after approximately $360^{\circ }$. This can be explained by the fact that in these areas there is no or only a slight orientation force (due to shear). These are the domains into which the oriented fibres flow after passing through the region around the interfering body. It can be determined that an orientation also takes place in areas far away from the cylinder (low shear), but this is much smaller than around the interfering body and thus show no significant effect. In the ‘Centre of Flowfield’ domain, it can be seen that the first eigenvalue and therefore the orientation does not increase significantly over the course of the entire experiment ($1440^{\circ }$). Only very low shear occurs here, which means that there is no/very little orientation of the fibres. It can also be seen that no ideal mixture can be guaranteed at the start of the experiment. However, the eigenvalue in the selected areas is in a good range between 0.55 and 0.65.

After rotating the container by 1440$^{\circ }$, no significant changes can be seen and $\lambda _{A1}$ remains in the range 0.75 and 0.8 for all selected areas. There is no area where a ‘perfect’ orientation can be measured ($\lambda _{A1} = 1$). This can be explained on the one hand by the fact that some fibres lie on the floor and therefore do not orientate themselves. Furthermore, the fibres move through a curved flow area. This inevitably results in different orientations in the same evaluation window, which limit the maximum value of $\lambda _{A1}$. In addition, the fixed resolution of the images means that a fixed number of pixels per fibre can be seen. This means that when calculating the angle with the binary gradient (see chapter "Analysis of the orientation"), the angle can only be determined with a certain degree of accuracy. This scattering of the angles can also be seen here. However, the fast orientation process can be clearly shown with the Figs 13 and 15.

## Conclusion and outlook

In this work, the fibre orientation of a flow around a cylinder in a rotating container with a transparent substitute liquid is analysed. In order to be able to sufficiently analyse the fibre orientation in the experiment, three basic approaches are developed:

A PIV measurement of the suspension is carried out. Small particles are introduced into the suspension, which are stimulated by a laser. This is used to determine a velocity vector field in a sectional plane using standard PIV measurement methods. A more precise analysis of the flow is carried out using the velocity gradient tensor $\textbf{L}$. This makes it possible to determine the flowtypes *f* in Fig. 9. Ultimately, these can be quantified in terms of strain rate $\dot{\varepsilon }$ and shear rate $\dot{\gamma }$, which provide information on the orientation effects in the flow region.

The suspension contains fibres that become visible when exposed to black light. The fibres are optically recorded and separated from the background using suitable CV and AI methods. By analysing the orientation of each pixel of the fibre, a detailed measurement of the orientation states is possible. Subsequently, an approach is chosen that establishes the second-level orientation tensor $\mathbf {A_2}$. Using suitable tensor analysis methods, the orientation can be represented in the entire flow area. See Fig. 13.

In order to be able to clearly separate the fibres from the background, a new approach to fibre tracking is used in this work. See Fig. 7 for the detailed procedure. A random forest classifier is used to detect fibres based on certain properties. Using a successive procedure (Fig. 7), it is sufficient to select 15 fibres on a training image for training. The trained random forest classifier can thus reliably detect fibres on the training image and also fibres on all other images of the measurement and further measurements. It can significantly outperform other methods (CNN, CV) due to its small amount of training data (15 fibres), the fast training time (a few seconds) and the fast application time (a few seconds per image).

Using these three methodological foundations, it is possible to carry out a precise analysis of the fibre orientation, which leads to the following results:

### PIV-measurement

The PIV measurements allow a reliable analysis of the flow domain, yielding velocity vector fields and streamlines that show low error levels over large areas of the field. Building on these data, a tensor-based evaluation of the velocity gradient and a flowtype analysis (Fig. 9) provide physically interpretable information about fibre-orienting effects. In particular, extensive regions of shear are identified that intensify in the vicinity of the interfering body and thereby drive fibre alignment along the local streamlines. Beneath the interfering body, a circular extensional flow with alternating compressive and elongational components is observed (Fig. 10). Consistent with earlier findings^39^, this pattern implies locally alternating orienting and de-orienting effects with respect to the streamlines. Far from the interfering body, the flow is predominantly rotational and in line with theory, does not show fibre orientation effects.

### Fibretracking

Fibre tracking with the random forest classifier proved effective and efficient. It robustly detects fibres with a very small training set and achieves both short training and inference times. A gradual, iterative selection of training data was used to minimise the required training data. By adding precisely those fibres that were previously missed or insufficiently recognised, the classifier generalised well across all recordings. For the present images, using intensity, gradient, and local binary pattern features was sufficient to obtain accurate segmentation without excessive computational cost. Occasional misclassifications were confined to areas with fibre-like reflections or strong edges in the background, where a small number of false positives or missed pixels can occur but do not affect the overall orientation statistics.

### Fibre orientation

The analysis in chapter "Evaluation of orientation" demonstrates that local fibre orientation can be quantified in detail using the second-level orientation tensor and its eigenstructure. Overall, the orientation evolves rapidly: fibres entering high-shear regions near the interfering body align along the local streamlines and a fully developed state is reached after approximately one full rotation of the container (Fig. 15). A strong alignment is also visible upstream of the interfering body. However, this region requires an additional rotation before the observed orientation stabilises. In contrast, fibres travelling through the low-shear area in the middle of the container show little or no alignment even after four rotations, which is consistent with the flow analysis in chapter "Determination of strain and shear rates".

The initial condition is close to isotropic over large areas. However, near the container walls and between the interfering body and the outer cylinder, fibre migration and wall interactions prevent a perfectly random initial orientation. Taken together, the measured orientation fields corroborate the expected influence of the local flowtype–shear, extensional, and rotational components as identified in chapter "Determination of strain and shear rates".

### Outlook

The present results establish a conclusive experimental pipeline for determining fibre orientation in complex creeping flows. Several targeted extensions can further improve accuracy and broaden applicability. First, illumination should be refined to reduce shadows and overexposed regions that arise from refractive-index mismatches between Plexiglas, silicone oil, and air. Second, the current random forest masks can be used to generate high-quality annotations for training a convolutional neural network. Additionally, separating individual fibres would allow explicit object tracking across frames and a direct analysis of single-fibre kinematics. Also near-wall measurements with higher spatial resolution are needed to quantify forced alignment and migration due to wall–fibre interactions, which influence the initial and local orientation states.

As the experiments were completed after four rotations, longer-duration experiments would help distinguish the transition between strongly aligned regions and the largely isotropic core and clarify slow orientation processes in low-shear zones. Developing quantitative models that map local shear and strain to orientation rates would enable predictive use of the measured flowtype fields. Finally, extending the measurements to three dimensions would resolve the out-of-plane orientation component and better characterise bottom effects in the current setup.

## Data Availability

The raw data of this article will be made available by the authors on request. You can contact Tim Vaupel (t.vaupel@uni-kassel.de) for this.

## Code availability

The code used in this article will be made available by the authors on request.
